# The Host Factor Early Growth Response Gene (EGR-1) Regulates *Vaccinia virus* Infectivity during Infection of Starved Mouse Cells

**DOI:** 10.3390/v10040140

**Published:** 2018-03-21

**Authors:** Leonardo C. de Oliveira, Bruno S. A. F. Brasil, Bethany Unger, Giliane S. Trindade, Jonatas S. Abrahão, Erna G. Kroon, Paula Traktman, Cláudio A. Bonjardim

**Affiliations:** 1 Grupo de Transdução de Sinal, Instituto de Ciências Biológicas, Universidade Federal de Minas Gerais, 31270-901 Belo Horizonte, Minas, Brazil; ldeolive2@gmail.com (L.C.d.O.); brunosafb@gmail.com (B.S.A.F.B.); 2 Laboratório de Vírus, Department of Microbiology, Institute of Biological Sciences, Universidade Federal de Minas Gerais, 31270-901 Belo Horizonte, Minas Gerais, Brazil; gitrindade@yahoo.com.br (G.S.T.); jonatas.abrahao@gmail.com (J.S.A.); ernagkroon@gmail.com (E.G.K.); 3 Department of Microbiology & Immunology, Medical College of Wisconsin, Milwaukee, WI 53226, USA; bugs@mcw.edu; 4 Departments of Biochemistry & Molecular Biology and Microbiology & Immunology, Medical University of South Carolina, Charleston, SC 29425, USA; traktman@musc.edu

**Keywords:** early growth response-1 (EGR-1), mitogen-activated protein kinase (MAPK), *Vaccinia virus*, virus–host cell interaction

## Abstract

Evolution has equipped poxvirus genomes with the coding capacity for several virus-host interaction products which interfere with host cell gene expression and protein function, creating an adequate intracellular environment for a productive infection. We show here that *Vaccinia virus* (VACV) induces the expression of the cellular transcription factor EGR-1 (early growth response-1) in Mouse Embryonic Fibroblasts (MEFs) through the MEK (mitogen-activated protein kinase (MAPK)/ERK)/ERK (extracellular signal-regulated kinases) pathway, from 3 to 12 h post infection (h.p.i.). By using starved *egr-1* knockout (*egr-1^−/−^*) MEFs, we demonstrate that VACV replication is reduced by ~1 log in this cell line. Although western blotting and electron microscopy analyses revealed no difference in VACV gene expression or morphogenesis, the specific infectivity of VACV propagated in *egr-1^−/−^* MEFs was lower than virus propagated in wild type (WT) cells. This lower infectivity was due to decreased VACV DNA replication during the next cycle of infection. Taken together, these results revealed that EGR-1 appears to facilitate VACV replication in starved fibroblasts by affecting viral particles infectivity.

## 1. Introduction

The *Poxviridae* family comprises large DNA viruses that have linear double-stranded genomes and which carry out their entire life cycle within the cytoplasmic compartment of the infected cell [1,2]. Members of this family of viruses have been extensively studied in the last few years aiming at creation of effective poxvirus vector-based vaccines as well as oncolityc vectors [3,4]. Although the World Health Organization declared smallpox eradicated in May 1980, the emergence of zoonotic *Vaccina virus* (VACV) in Brazil and its association with the disease known as bovine vaccinia [5], more studies, nonetheless, are required to better understand VACV-host interactions. *Vaccinia virus* (VACV) is the prototypic poxvirus with a ~200 Kb genome and can infect a broad range of hosts, including rodents and humans [2,6]. About half of VACV genome codes for virus-host interaction products that are implicated in both evading host immune defenses and in creating favorable replicative conditions within cells and tissues [7,8]. Among these are virokines, viroceptors and viral growth factors that act extracellularly, as well as viral proteins that interfere with intracellular signaling pathways [8,9,10]. An example of the former is the Vaccinia growth factor (VGF), a polypeptide that shares sequence and functional similarity to cellular growth factors and is secreted from VACV infected cells to stimulate a proliferative response in surrounding cells [11,12].

The MAPK (Mitogen-Activated Protein Kinase) signaling cascade is a major pathway associated with the transmission of mitogenic and survival signals in response to a variety of extracellular stimuli [13]. Indeed, VACV infection of murine fibroblasts induces the activation of MAPK/ERK1/2 (extracellular-signal-regulated kinase 1/2), which occurs partially through the action of VGF [10,14,15]. This activation appears to facilitate viral replication as inhibition of this pathway has been shown to lead to a large reduction in viral growth [15]. Interestingly, while infection of murine fibroblasts with the rabbit specific leporipoxvirus MYXV (*Myxoma virus*) can also stimulate ERK1/2 phosphorylation, the activation of this pathway renders these cells non-permissive for virus infection through the activation of the transcription factor IRF-3 (Interferon regulatory factor 3) and upregulation of IFN-β (β-Interferon) expression [9,16].

The cellular transcription factor EGR-1 (early growth response-1) was identified as a downstream target of MEK (mitogen-activated protein kinase (MAPK)/ERK)/ERK (extracellular signal-regulated kinases) that is activated during both VACV and CPXV infection [17]. However, this factor appears to play a distinct role during the infections of these two *Orthopoxviruses* as depletion of EGR-1 by siRNA solely affects VACV replication [16]. EGR-1 is an 82 kDa phosphoprotein with a modular structure, containing both a DNA-binding domain equipped with three C2H2 zinc fingers that bind to the consensus DNA sequence 5-GCG(G/T)GGGCG-3 [18] and an activation/repression domain, a structure that is consistent with the diverse activities associated with the molecule [19,20]. EGR-1 couples extracellular stimulation elicited by growth factors, cytokines and environmental stress to cellular responses associated with proliferation, apoptosis and tissue injury [19,21]. Moreover, EGR-1 has recently been implicated in neuronal plasticity and memory formation [22,23], as well as cancer invasion, by upregulating the expression of cysteine protease cathepsin D [24].

Different viruses, such as the KSHV (Kaposi’s sarcoma-associated herpesvirus), HSV-1 (herpes simplex virus type-1), JCV (human polyomavirus JC virus), HIV (human immunodeficiency virus) and EBV (Epstein-Barr virus) are also capable of modulating the *egr-1* gene function during infection [25,26,27,28,29].

We show here that deletion of the *egr-1* gene does not affect VACV gene expression or morphogenesis but diminishes the specific infectivity of the virus particles produced in starved cells.

## 2. Materials and Methods

### 2.1. Cell Culture, Antibodies and Chemicals

Wild type (WT) and *egr-1* knockout (*egr-1^−/−^*) mouse embryonic fibroblasts (MEFs) from the same genetic background were kindly provided by Dr. Eileen D. Adamson (USA) [30]. The cells were cultured in Dulbecco’s modified Eagle’s medium (DMEM), supplemented with 7% (*v*/*v*) heat-inactivated fetal bovine serum (FBS) and antibiotics in 5% CO_2_ at 37 °C. Cells were starved after reaching 90% confluence by changing the medium to 0.5% FBS for 12 h. The Rhesus monkey kidney fibroblast cell line BSC-40 (ATCC CRL-2761) was cultured in modified Eagle’s medium (MEM), supplemented with 7% (*v*/*v*) (FBS) and antibiotics in 5% CO_2_ at 37 °C. The commercially available antibodies and chemicals used were: rabbit polyclonal antibody against EGR-1 (Santa Cruz Biotech, Dallas, TX, USA), mouse monoclonal anti-β-actin antibody, peroxidase-conjugated anti-rabbit secondary antibody peroxidase-conjugated anti-mouse secondary antibody and the MEK inhibitor U0126 (Cell Signaling, Danvers, MA, USA). The antibodies against the viral proteins A13, D8, H3, F18, A17, p4b/4b and B14 (SPI-2/crmA) were provided by Dr. Bernard Moss (NIAID, Bethesda, MD, USA). The MEK inhibitor U0126 was used at a concentration of 15 µM.

### 2.2. Virus Infection and Purification

Wild type VACV, strain WR, was propagated into BSC-40 cells and purified by sucrose gradient sedimentation as previously described [31]. Infectivity of the purified viruses was determined by the plaque assay in BSC-40 cells. The total number of particles was determined from the OD at 260 nm by using the following formula: 1 OD = 1.2 × 10^10^ particles [32] and confirmed by Bradford analysis of protein content at 595 nm and by real time polymerase chain reaction (PCR) analysis. The experiments presented in this study were carried out with the mature virion (MV) form of VACV. Before infection, cells were cultured until they reached 80–90% confluence and were subsequently serum starved (0.5% FBS, for 12 h). The cells were infected at the indicated multiplicity of infection (MOI) for the times shown.

### 2.3. Virus Infectivity Assays

WT or *egr-1^−/−^* MEFs were cultured, serum deprived or not, at a density of 5 × 10^5^ cells per well, on a 6-well culture dish and then VACV-infected. Infections were carried out at an MOI of 10 for 3, 6, 12, 24, 36 and 48 h. Where indicated, cells were incubated with the MEK pharmacological inhibitor U0126 for 30 min prior to infection, which was kept in the media throughout the infection.

Virus present in the disrupted cell lysates was assayed for infectivity. The 48 h.p.i. time-point was run in triplicate and the results represent the average values. Statistical analysis by two-tailed Student’s *T*-test was performed with the ABI prism 3.0 software (La Jolla, CA, USA).

### 2.4. Western Blotting

#### 2.4.1. Whole-Cell Lysate Preparation

WT or *egr-1^−/−^* MEFs were grown and starved as above and infected with VACV at an MOI of 5 or 10 for the indicated times. Cells were left untreated or were pre-incubated for 30 min with U0126 when indicated, which remained throughout the infection. Cell lysates were generated as described previously [16].

#### 2.4.2. Electrophoresis and Immunoblotting

Thirty micrograms of the cell lysate per sample was separated on a 10% SDS-Acrylamide gel and transferred to nitrocellulose membranes. Membranes were blocked, washed and incubated with a specific rabbit polyclonal anti-EGR-1 (1:1500) or anti-viral proteins (1:3000) or mouse monoclonal anti-β-actin (1:3000) antibodies. Immunoreactive bands were visualized using the enhanced chemiluminescence (ECL) detection system (GE Healthcare, São Paulo, Brazil).

### 2.5. Real Time PCR

WT or *egr-1^−/−^* MEFs were serum starved or grown in media containing 7% serum and infected with VACV at an MOI of 10. Infections were carried out for 3, 6, 9 or 24h with DNA extraction at each time point through the phenol-chloroform procedure as described previously [33]. Viral load was determined using the primers (F 5′CTGTAGTTATAAACGTTCCGTGTG3′ and R 5′TTATCATACGCATTACCATTTCGA3′) to target the viral RNA polymerase subunit *rpo18* gene (D7R) [34] and the results were normalized using the primers designed for the cellular primate DNA for the IL12p40 subunit gene (F 5′GTAGAGGTGGACTGGACTCC3′ and R 5′CAGATGTGAGTGGGTCAGAG3′). A 5-fold serial dilution of VACV DNA extracted from 48 h infected cells was used as to construct a standard curve in order to validate the experiments. The parameters obtained were: VACV-rpo18 primers (Slope: −3.345; Efficiency: 98.99%; *R*^2^: 0.981) IL12p40 (Slope: −3.385; Efficiency: 97.44%; *R*^2^: 0.983). Viral load was quantified as arbitrary units relative to the reference: 1 = viral load at 3 h.p.i. in WT MEFs (∆∆*C*T method). PCR conditions were as follows: initial denaturation: 5 min at 95 °C followed by 30 cycles of: 30 s at 95 °C, 30 s at 58 °C, 30 s at 72 °C. Graphics and statistical analyses were performed by using ABI Prism 3.0 software (La Jolla, CA, USA).

### 2.6. Electron Microscopy

WT or *egr-1^−/−^* MEFs were maintained in low serum for 24 h and then infected with VACV at an MOI of 2 for 18 h. Cells were washed with phosphate buffered saline (PBS) and fixed in situ with 1% glutaraldehyde in PBS (pH 7.4) for 1 h at room temperature. Cells were then processed for conventional transmission electron microscopy and thin sections were examined on a Hitachi H-600 microscope (São Paulo, Brazil).

## 3. Results

### 3.1. EGR-1 Protein Expression Is Stimulated through the MEK/ERK Pathway upon VACV Infection and Is Required for Replication in Starved Fibroblasts

**Figure 1:**
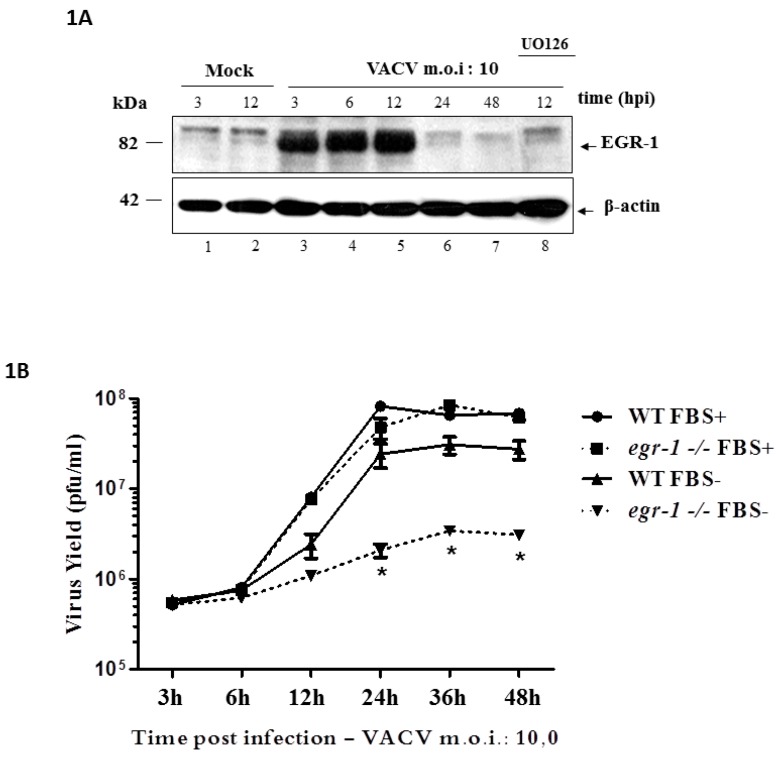
Expression of early growth response-1 EGR-1 protein during *Vaccina virus* (VACV) infection and effects of its deletion in viral replication. (**A**) VACV infection stimulates EGR-1 expression through the MEK (mitogen-activated protein kinase (MAPK)/ERK)/ERK (extracellular signal-regulated kinases) pathway with similar patterns—Western blotting analysis of VACV-infected wild type (WT) mouse embryonic fibroblasts (MEFs) at a multiplicity of infection (MOI) of 10 for the times indicated. Cells were starved with 0.1% fetal bovine serum (FBS) for 12 h and then were either Mock-infected or infected with VACV (lanes 3–8). Where indicated, cells were incubated for 30 min with the MEK inhibitor U0126 (15 µM) prior to and throughout the course of infection (lane 8). The molecular masses are indicated (KDa) on the left. (**B**) VACV growth curve performed with MEFs WT and *egr-1^−/−^*—Cells were infected with VACV at an MOI of 10 for 3, 6, 12, 24, 36 or 48 h. Infection occurred in the presence of 7% FBS (FBS+) (Cycling MEFs) or 0.5% FBS (FBS−) (starved MEFs). Cell-associated virus was then collected and titrated. Data is representative of three independent experiments also titrated in triplicates with similar results (*N* = 3, * = *p* < 0.001). Statistical analyses were performed by using ABI Prism 3.0 software.

This study aimed to address the VACV replication cycle step(s) affected by EGR-1 disruption. To that end, wild-type (WT) MEFs and those with an inactivated *egr-1* locus (*egr-1^−/−^*), generated by Virolle and collaborators [30] were used. Cells were deprived of FBS, that is, 0.1% FBS—12 h and then infected with VACV at an MOI of 10. Figure 1A shows that VACV infection stimulated the expression of EGR-1 protein in the WT MEFs through the MEK/ERK pathway. EGR-1 protein accumulation could be detected by western blotting (WB) from 3 to 12 h.p.i. (Figure 1A, lanes 1–7) and was abolished when the infection was carried out in the presence of the MEK inhibitor, U0126 (Figure 1A, lane 8). As a control, a WB showing the levels of EGR-1 in proliferating as compared to starved cells is shown (Supplementary Figure S1). Data concerning the proliferation rates of WT or *egr-1^−/−^* cells growing in the presence of 0.1% or 10% fetal bovine serum (FBS) is also provided (Supplementary Figure S2). In addition, VACV growth curve experiments showed that the yield of infectious VACV was decreased by ~1 log when the infection was carried out in serum-starved (FBS−) *egr-1^−/−^* MEFs rather than WT MEFs, as shown in Figure 1B. The same reduction was not observed in VACV yield when the infection was carried out in cycling (not serum-deprived—FBS+) *egr-1^−/−^* MEFs (Figure 1B), suggesting that EGR-1 is required for VACV replication particularly in the intracellular environment of starved cells.

### 3.2. EGR-1 Gene Deletion Does Not Affect VACV Gene Expression or Morphogenesis in MEFs

Next, we designed a set of experiments to identify which step of the VACV replication cycle was affected by *egr-1* gene deletion in starved MEFs. Several studies have implicated host cell proteins, such as TBP—TATA-binding protein and G3BP/Caprin 1, in VACV intermediate/late transcription in vitro [35,36,37,38,39,40], therefore we asked whether VACV gene expression would be altered in *egr-1^−/−^* MEFs.

**Figure 2:**
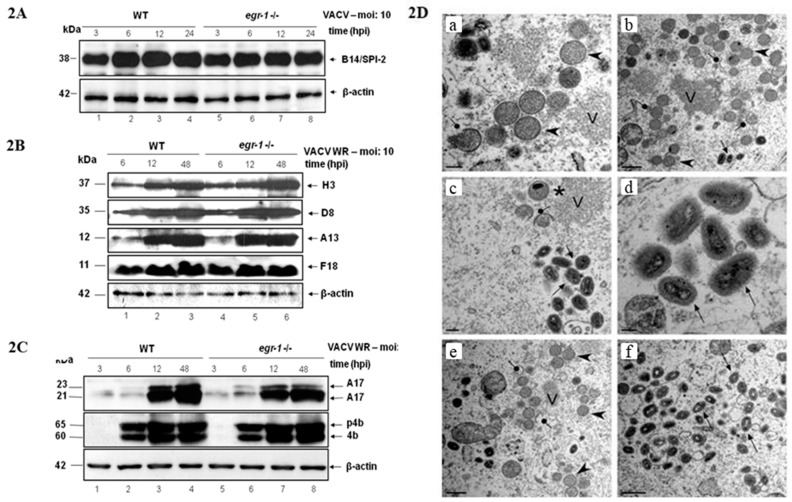
*Egr-1* gene deletion does not affect VACV gene expression, protein processing or viral morphogenesis. (**A**–**C**) *Egr-1* gene deletion does not affect VACV gene expression or protein processing—Western blotting analysis of VACV infected WT and *egr-1^−/−^* starved MEFs (FBS 0.1%) at an MOI of 10 for the indicated times and with the following antibodies: ((**2A**)—Early viral gene expression) anti-B14 antibody; ((**2B**)—Late viral gene expression) anti-H3, A13, F18 and D8 antibodies; ((**2C**)—Viral protein processing) anti-A17 and -p4b/4b antibodies. The molecular masses are indicated (KDa) on the left. (**D**) *Egr-1* gene deletion does not affect VACV morphogenesis—Confluent monolayers of WT and *egr^−/−^* MEFs were serum-starved for 12 h and infected with VACV for 18 h at an MOI of 2. Cells were then fixed in situ and processed for transmission electron microscopy. WT (panels e,f) and *egr^−/−^* MEFs (panels a–d) contained all the normal intermediates in virion morphogenesis. Simbols—arrowheads: Immature virions; V: Virosomes; lollipops: Crescents; arrows: Mature virions; asterisk: Immature virions with nucleoids; Mature virions with proper brick-shaped morphology and biconcave core: arrows (panel d). Scale bar: a,c—200 nm. b,e,f—400 nm. d—100 nm. Data are representative of three independent experiments with similar results.

Analysis of WT and *egr-1^−/−^* MEFs infected with VACV under low serum conditions did not reveal any difference in viral gene expression as demonstrated by western blot analyses of either the early protein B14/SPI-2 (Figure 2A) or the late proteins H3, A13, F18 and D8 (Figure 2B). The same results were also verified with the early protein H5, the early/intermediate protein I3 and the late proteins A14. The processing of the viral proteins A17 and p4b/4b (Figure 2C) was unaffected by the absence of EGR-1.

Since *egr-1* gene deletion affects the yield of infectious virus without impairing viral gene expression, we next asked whether virion morphogenesis could be impaired by the absence of EGR-1. WT and *egr-1^−/−^* MEFs were serum starved and subsequently infected with VACV at an MOI of 2 for 18 h, after which the cells were processed for transmission electron microscopy. The full spectrum of virion morphogenesis intermediates was observed in both WT (Figure 2D—panels E–F)) and *egr-1^−/−^* cells (Figure 2D—panels A–D). Virosomes (V, panel A–C and E), membrane crescents (lollipops, panels B,C,E), immature virions (arrowheads, panels A,B,E), immature virions with nucleoids (asterisk, panel C) and mature virions (arrow, panels B–D and F) were seen in abundance. Wrapped virions, which are the precursor of the small pool of enveloped virions that are released from infected cells by exocytosis, were also seen.

### 3.3. VACV Produced in egr-1^−/−^ MEFs Have Reduced Specific Infectivity

**Figure 3:**
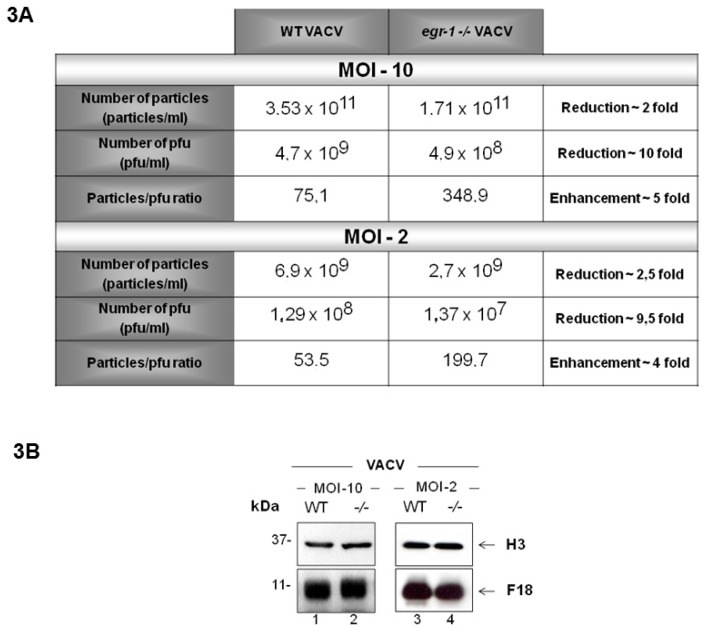
VACV formed in *egr-1^−/−^* MEFs presents decreased infectivity. (**A**) Table showing the particle:pfu ratio (specific infectivity) of VACV propagated in WT and *egr-1^−/−^* MEFs. VACV was propagated in serum starved WT and *egr-1^−/−^* MEFs at an MOI of 10 or 2. After 48 h.p.i. virus was collected and purified. Infectivity of the purified viruses was determined by the plaque assay in BSC-40 cells. The total number of particles was determined from the OD at 260 nm by using the formula 1 OD = 1.2 × 10^10^ particles [32] and was confirmed by both the Bradford analysis of protein content at 595 nm and by real time PCR. Data present the mean values of two independent experiments, with each MOI; (**B**) western blot analysis of VACV particles propagated in WT and *egr-1^−/−^* MEFs. Equivalent amounts of WT VACV and *egr-1^−/−^* VACV particles were submitted to SDS-PAGE and immunoblotting with anti-H3 and anti-F18 antibodies to corroborate spectrophotometric quantification of virus particles. Data are representative of two independent experiments, with each MOI, with similar results.

Although the yield of infectious VACV in serum-starved *egr-1^−/−^* MEFs was reduced by ~1 log as compared to what we observed in WT MEFs, (neither the biochemical progression of the life cycle nor virion morphogenesis appeared to be impaired). It seemed possible, that virions formed in the absence of EGR-1 might be decreased in their infectivity. In order to address this question VACV was propagated in serum-starved *WT* or *egr-1^−/−^* MEFs at an MOI of 10. To confirm that the same phenotypes observed during VACV infection of *egr-1^−/−^* MEFs with an MOI of 10 occurred under the conditions optimized for electron microscopy analysis (MOI of 2, 18 h.p.i.), these experiments were also performed at an MOI of 10. At 24 h.p.i., cells were collected and the virus particles were purified by sucrose gradient sedimentation. The particle:pfu ratio of purified VACV propagated in *egr-1^−/−^* MEFs (VACV *egr-1^−/−^*) was 4- to 5-fold higher than that of VACV propagated in WT MEFs (VACV WT) (Figure 3A). These results confirm the hypothesis that VACV *egr-1^−/−^* particles possess a decreased level of specific infectivity. A minor 2-fold reduction in the total number of particles produced in *egr-1* knockout cells was also observed (Figure 3A). Figure 3B shows western blot analysis of the mature virions (MV) structural proteins H3 and F18 present in equivalent amounts of purified VACV WT and VACV *egr-1^−/−^* particles propagated at MOI 2 and 10. This assay corroborates the quantification of total virus particles obtained by spectrophotometric analysis and real time PCR shown in Figure 3A.

### 3.4. VACV Produced in MEFs egr-1^−/−^ Presents Delayed Viral DNA Replication during the Next Round of Infection

To determine at which stage(s) of virus replication the reduced infectivity would become evident, BSC-40 cells were infected in parallel with equal numbers of VACV produced in WT and *egr-1^−/−^* MEFs.

**Figure 4:**
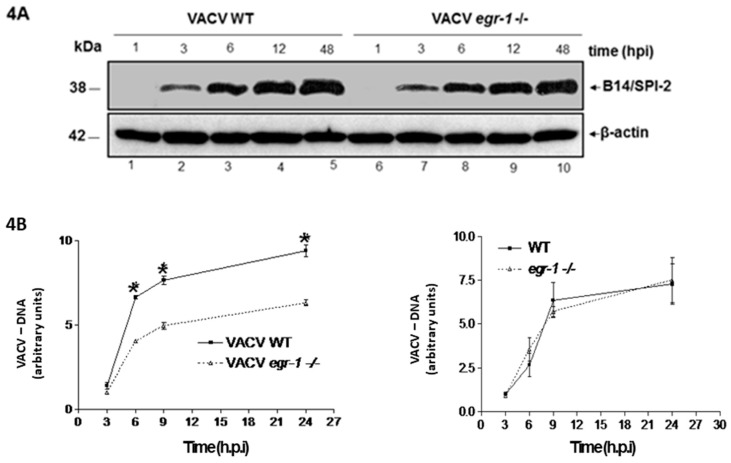
VACV formed in *egr-1^−/−^* MEFs presents delayed viral DNA replication. (**A**)—VACV produced in *egr-1^−/−^* MEFS exhibit no defect in viral early gene expression. Western blotting analysis of BSC-40 cells infected with VACV WT at an MOI of 5 or the corresponding amount of VACV propagated into *egr-1^−/−^* for the indicated times. (**A**)—The membrane was probed with the anti-viral protein antibodies: B14/SPI 2. Bottom panels—Anti β-actin antibody was used as a loading control. Data are representative of three independent experiments. (**B**)—VACV from *egr-1^−/−^* MEFs presents defects in viral DNA replication. Graphic representation of viral DNA accumulation in BSC-40 cells infected with VACV at an MOI of 5 propagated in WT or the corresponding amount of VACV propagated in *egr-1^−/−^* cells as determined by quantitative real time PCR analysis (left panel). The same experiment was also performed with VACV produced in WT or *egr-1^−/−^* cells that were grown in the presence of 7% FBS (right panel). Viral load was quantified as arbitrary units relative to the reference: 1= viral load at 3 h.p.i. in WT MEFs (∆∆*C*_T_ method). A 5-fold serial dilution of VACV DNA extracted from 48 h infected cells was used as to construct a standard curve in order to validate the experiments. The parameters obtained were: VACV-rpo18 primers (Slope: −3.345; Efficiency: 98.99%; *R*^2^: 0.981) IL12p40 (Slope: −3.385; Efficiency: 97.44%; *R*^2^: 0.983). Data are representative of three independent experiments (*N* = 3, * = *p* < 0.001). Statistical analyses were performed by using ABI Prism 6.0 software.

BSC-40 were grown in 7% FBS and infected with VACV WT at an MOI of 5.0 or with the corresponding number of VACV *egr-1^−/−^* particles. Viral protein expression and processing (Figure 4A) and viral DNA replication (Figure 4B) were then analyzed at various times post-infection. Figure 4A shows that the expression levels of the early protein B14/SPI-2 were similar regardless if the infections were carried out with VACV that had been propagated in *egr-1^−/−^* or in WT MEFs. The kinetics of protein expression showed similar patterns of protein expression (Figure 4A). By contrast, a statistically significant decrease in the levels of replicated viral DNA was seen in cells infected with VACV propagated in *egr-1^−/−^* MEFs as compared to those infected with VACV propagated in WT MEFs (Figure 4B—left panel). As a control, we also compared the VACV DNA yield with virus produced in WT or *egr-1^−/−^* cells in the presence of 7% FBS. Our result clearly showed that, under this circumstance, there is no difference in the levels of replicating viral DNA (Figure 4B—right panel). Taken together, our results indicate that the decreased infectivity observed with VACV particles produced in starved *egr-1^−/−^* MEFs may be due to defects in DNA replication that do not appear to be a consequence of decreased availability of early viral proteins.

## 4. Discussion

Host-pathogen relationships provide powerful evolutionary forces that shape biological diversity [41]. Throughout coevolution with their hosts, *orthopoxvirus* genomes have acquired the coding capacity for several gene products that modulate host cell function and therefore act as key determinants of viral tropism and pathogenesis [6,42]. For instance, during VACV infection, the viral protein VGF binds to its cellular receptor EGF and triggers mitogenic signaling cascades, a process that results in the transcriptional activation of cellular genes and promotes cell cycle progression [9,42,43,44]. The action of VGF increases viral replication in quiescent cells in vitro [10,11], synergizes with F1L in the inhibition of apoptosis during VACV infection [45] and enhances viral biodistribution and virulence in vivo [46].

It has been demonstrated that mitogenic stimulation creates a favorable environment for the replication of several viruses such as papillomaviruses and adenoviruses. Poxviruses also display greater infectivity in cycling cells (Figure 1B; reviewed in [8]). We have previously shown that VACV infection—partially through the action of VGF—leads to MAPK pathway activation and the consequent expression of the cellular genes *c-fos* and *egr-1* [13,14]. Furthermore, activation of the MEK/ERK/EGR-1 pathway facilitates specifically VACV replication in the A31 cell line as demonstrated by siRNA gene targeting against EGR-1 [16]. In this study, we demonstrate that infection of WT MEFs by VACV stimulates a similar pattern of EGR-1 expression through the MEK/ERK pathway (Figure 1A). However, while EGR-1 expression in MEFs (Figure 1A) ceases earlier than in A31 cells, loss of function of EGR-1 had the same impact on VACV replication as previously reported [16], since *egr-1* gene deletion decreased VACV growth by ~1 log (Figure 1B), a phenomenon restricted to serum-starved mouse cells (Figure 1B). Whether the decrease in EGR-1 expression reflects targeting of the protein for degradation, transcription turning off or both remains an issue to be pursued further. Thus, in line with the fact that EGR-1 is a downstream target of VGF [14] our data suggest that this cellular transcription factor is required specifically in the intracellular environment of starved cells.

Several studies have implicated the cellular transcription factors SP1, YY1 and TBP, as well as the RNA binding proteins G3BP/Caprin-1 in VACV intermediate/late transcription in vitro [35,36,38,47]. These host cell proteins were also shown to colocalize with VACV DNA factories during infection [35,39,40]. Indeed, VACV post-replication transcription complexes require cellular factors for efficient transcription [48,49,50]. This does not seem to be the case for EGR-1 though, since we were unable to detect any interaction of EGR-1 with intermediate/late viral promoter sequences and viral transcriptional complexes by EMSA. Analysis of the subcellular localization of EGR-1 during VACV infection also revealed a nuclear restricted localization of the protein [16]. In addition, the results presented in Figure 2A and Figure 2B do not provide evidence for EGR-1 participation in VACV gene expression, since neither early (Figure 2A) nor intermediate/late (Figure 2B) protein expression was affected in starved *egr-1^−/−^* MEFs.

Further analysis revealed that neither VACV proteolytic processing of late proteins (Figure 2C) nor morphogenesis (Figure 2D) was impacted in *egr-1^−/−^* MEFs. All the stages of viral morphogenesis were observed, at comparable abundance, in both WT and *egr-1^−/−^* MEFs (Figure 2D) (Reviewed in [1,2]). In line with these findings, as shown in Figure 3A, VACV particles produced in starved *egr-1^−/−^* MEFs had a 4–5-fold reduction in their specific infectivity, with only a slight (~2-fold) reduction in the total number of particles (Figure 3A). Taken together, these results indicate that EGR-1 deletion has little effect on VACV particle formation but it appears to impact the infectivity of the viral particles formed.

We further investigated the progression of a subsequent round of infection performed in BSC40 cells with equal numbers of VACV particles produced in starved WT or *egr-1^−/−^* MEFs (Figure 4A,B). While viral early protein expression appeared to proceed normally in cells infected with VACV *egr-1^−/−^* particles (Figure 4A), the accumulation of viral DNA (Figure 4B). It is important to note that the EGR-1 protein cannot be directly responsible for this phenotype as EGR-1 was not found in purified WT VACV particles by either western blot analysis or mass spectrometry [51,52]. In addition, EGR-1 protein was no longer detectable in cell extracts from infected cells at later times during infection (Figure 1A). It has been shown that VACV encoded uracil DNA glycosylase and dUTPase activities are required for normal viral DNA replication in the intracellular environment of quiescent cells [53]. Studies conducted by Mo and collaborators [44] reveal that *Orf virus* regulates cell cycle progression to S phase while repressing progression through G2/M, creating a supportive intracellular environment for viral genome replication. Although the mechanism through which EGR-1 facilitates VACV replication remains elusive, we hypothesize that this cellular protein might exert a role in reprogramming gene expression in quiescent cells during viral infection. The observation that VACV *egr-1^−/−^* particles express early proteins in similar patterns as WT particles (Figure 4A) suggests that the DNA encapsidated in VACV propagated into *egr-1^−/−^* cells might be itself in some way altered. In line with this observation our previous data demonstrated that EGR-1 expression is regulated by the transcription factor JUN through the binding of JUN to the AP-1 sequence found at the promoter region of *egr-1.* Moreover, viral DNA synthesis was affected in cell line expressing c-Jun dominant-negative mutation [54]. We conclude, based on the fact that JUN expression at times where viral DNA is synthesized [55] is governed by the MEK/ERK pathway [13,14,54] and that the MEK/ERK/JUN/EGR pathway is required for maximal viral DNA synthesis. This may reflect an altered conformation that impairs proper DNA replication, or it may result from the absence of an as yet unknown encapsidated factor that aids replication. However, future studies aiming to address these issues are required.

Our findings reveal VACV specific adaptations for optimal replication in a starved intracellular environment. It would also be of interest to evaluate the tropism and oncolytic potential of recombinant *Orthopoxviruses* expressing dominant-negative forms for EGR-1 protein in vivo.

## Supplementary Materials

Supplementary materials can be found at http://www.mdpi.com/1999-4915/10/4/140/s1.

## References

[1] Condit R.C., Moussatche N., Traktman P. (2006). In a nutshell: Structure and assembly of the vaccinia virion. Adv. Virus Res..

[2] Moss B., Knipe D.M., Howley P.M. (2013). Poxviridae. Fields Virology.

[3] Fukuhara H., Ino Y., Todo T. (2016). Oncolytic virus therapy: A new era of cancer treatment at dawn. Cancer Sci..

[4] Haddad D. (2017). Genetically Engineered Vaccinia Viruses as Agents for Cancer Treatment, Imaging and Transgene Delivery. Front. Oncol..

[5] Oliveira J.S., Figueiredo P.O., Costa G.B., Assis F.L., Drumond B.P., da Fonseca F.G., Nogueira M.L., Kroon E.G., Trindade G.S. (2017). Vaccinia Virus Natural Infections in Brazil: The Good, the Bad and the Ugly. Viruses.

[6] Lefkowitz E.J., Wang C., Upton C. (2006). Poxviruses: Past, present and future. Virus Res..

[7] Johnston J.B., McFadden G. (2004). Technical knockout: Understanding poxvirus pathogenesis by selectively deleting viral immunomodulatory genes. Cell Microbiol..

[8] Smith G.L., Benfield C.T.O., de Motes C.M., Mazzon M., Ember S.W.J., Ferguson B.J., Sumner R.P. (2013). Vaccinia virus immune evasion: Mechanisms, virulence and immunogenicity. J. Gen. Virol..

[9] McFadden G. (2005). Poxvirus tropism. Nat. Rev. Microbiol..

[10] Bonjardim C.A. (2017). Viral exploitation of the MEK/ERK pathway—A tale of vaccinia virus and other viruses. Virology.

[11] Twardzik D.R., Brown J.P., Ranchalis J.E., Todaro G.J., Moss B. (1985). Vaccinia virus-infected cells release a novel polypeptide functionally related to transforming and epidermal growth factors. Proc. Natl. Acad. Sci. USA.

[12] Brown J.P., Twardzik D.R., Marquardt H., Todaro G.J. (1985). Vaccinia virus encodes a polypeptide homologous to epidermal growth factor and transforming growth factor. Nature.

[13] Yang S.H., Sharrocks A.D., Whitmarsh A.J. (2013). MAP kinase signalling cascades and transcriptional regulation. Gene.

[14] De Magalhães J.C., Andrade A.A., Silva P.N., Sousa L.P., Ropert C., Ferreira P.C., Kroon E.G., Gazzinelli R.T., Bonjardim C.A. (2001). A mitogenic signal triggered at an early stage of vaccinia virus infection: Implication of MEK/ERK and protein kinase A in virus multiplication. J. Biol. Chem..

[15] Andrade A.A., Silva P.N., Pereira A.C., de Sousa L.P., Ferreira P.C., Gazzinelli R.T., Kroon E.G., Ropert C., Bonjardim C.A. (2004). The vaccinia virus-stimulated mitogen-activated protein kinase (MAPK) pathway is required for virus multiplication. Biochem. J..

[16] Wang F., Ma Y., Barrett J.W., Gao X., Loh J., Barton E., Virgin H.W., McFadden G. (2004). Disruption of Erk-dependent type I interferon induction breaks the myxoma virus species barrier. Nat. Immunol..

[17] Silva P.N., Soares J.A., Brasil B.S., Nogueira S.V., Andrade A.A., de Magalhães J.C., Bonjardim M.B., Ferreira P.C., Kroon E.G., Bruna-Romero O. (2006). Differential role played by the MEK/ERK/EGR-1 pathway in Orthopoxviruses vaccinia and Cowpox biology. Biochem. J..

[18] Thiel G., Cibelli G. (2002). Regulation of life and death by the zinc finger transcription factor Egr-1. J. Cell. Physiol..

[19] Liu C., Rangnekar V.M., Adamson E., Mercola D. (1998). Suppression of growth and transformation and induction of apoptosis by EGR-1. Cancer Gene Ther..

[20] Sukhatme V.P., Cao X.M., Chang L.C., Tsai-Morris C.H., Stamenkovich D., Ferreira P.C., Cohen D.R., Edwards S.A., Shows T.B., Curran T. (1988). A zinc finger-encoding gene coregulated with c-fos during growth and differentiation and after cellular depolarization. Cell.

[21] Silverman E.S., Collins T. (1999). Pathways of Egr-1-mediated gene transcription in vascular biology. Am. J. Pathol..

[22] Veyrac A., Besnard A., Caboche J., Davis S., Laroche S. (2014). The transcription factor Zif268/Egr1, brain plasticity and memory. Prog. Mol. Biol. Transl. Sci..

[23] Duclot F., Kabbaj M. (2017). The Role of Early Growth Response 1 (EGR1) in Brain Plasticity and Neuropsychiatric Disorders. Front. Behav. Neurosci..

[24] Park Y.J., Kim E.K., Bae J.Y., Moon S., Kim J. (2016). Human telomerase reverse transcriptase (hTERT) promotes cancer invasion by modulating cathepsin D via early growth response (EGR)-1. Cancer Lett..

[25] Dyson O.F., Traylen C.M., Akula S.M. (2010). Cell Membrane-bound Kaposi’s Sarcoma-associated Herpesvirus-encoded Glycoprotein B Promotes Virus Latency by Regulating Expression of Cellular Egr-1. J. Biol. Chem..

[26] Hsia S.C., Graham L.P., Bedadala G.R., Balish M.B., Chen F., Figliozzi R.W. (2013). Induction of Transcription Factor Early Growth Response Protein 1 during HSV-1 Infection Promotes Viral Replication in Corneal Cells. Br. Microbiol. Res. J..

[27] Romagnoli L., Sariyer I.K., Tung J., Feliciano M., Sawaya B.E., Del Valle L., Ferrante P., Khalili K., Safak M., White M.K. (2009). Early growth response-1 protein is induced by JC virus infection and binds and regulates the JC virus promoter. Virology.

[28] Fan Y., Zou W., Green L.A., Kim B.O., He J.J. (2011). Activation of Egr-1 expression in Astrocytes by HIV-1 Tat: New Insights into Astrocyte-Mediated Tat Neurotoxicity. J. Neuroimmune Pharmacol..

[29] Vockerodt M., Wei W., Nagy E., Prouzova Z., Schrader A., Kube D., Rowe M., Woodman C.B., Murray P.G. (2013). Suppression of the LMP2A target gene, EGR-1, protects Hodgkin's lymphoma cells from entry to the EBV lytic cycle. J. Pathol..

[30] Virolle T., Adamson E.D., Baron V., Birle D., Mercola D., Mustelin T., de Belle I. (2001). The Egr-1 transcription factor directly activates PTEN during irradiation-induced signalling. Nat. Cell Biol..

[31] Joklik W.K. (1962). The purification of four strains of poxvirus. Virology.

[32] Earl P.L., Moss B., Wyatt L.S., Carroll M.W., Ausubel F.M., Brent R., Kingston R.E., Moore D.D. (1998). Current Protocols in Molecular Biology.

[33] Sambrook J., Fritsch E.F., Maniats T. (1989). Molecular Cloning: A Laboratory Manual.

[34] Olson V.A., Laue T., Laker M.T., Babkin I.V., Drosten C., Shchelkunov S.N., Niedrig M., Damon I.K., Meyer H. (2004). Real-time PCR system for detection of orthopoxviruses and simultaneous identification of smallpox virus. J. Clin. Microbiol..

[35] Broyles S.S., Liu X., Zhu M., Kremer M. (1999). Transcription factor YY1 is a vaccinia virus late promoter activator. J. Biol. Chem..

[36] Katsafanas G.C., Moss B. (2004). Vaccinia virus intermediate stage transcription is complemented by Ras-GTPase-activating protein SH3 domain-binding protein (G3BP) and cytoplasmic activation/proliferation-associated protein (p137) individually or as a heterodimer. J. Biol. Chem..

[37] Oh J., Broyles S.S. (2005). Host cell nuclear proteins are recruited to cytoplasmic vaccinia virus replication complexes. J. Virol..

[38] Knutson B.A., Liu X., Oh J., Broyles S.S. (2006). Vaccinia virus intermediate and late promoter elements are targeted by the TATA-binding protein. J. Virol..

[39] Katsafanas G.C., Moss B. (2007). Colocalization of transcription and translation within cytoplasmic poxvirus factories coordinates viral expression and subjugates host functions. Cell Host Microbe.

[40] Simpson-Holley M., Kedersha N., Dower K., Rubins K.H., Anderson P., Hensley L.E., Connor J.H. (2011). Formation of antiviral cytoplasmic granules during Orthopoxvirus infection. J. Virol..

[41] Burdon J.J., Thrall P.H., Ericson L. (2013). Genes, communities & invasive species: Understanding the ecological and evolutionary dynamics of host-pathogen interactions. Curr. Opin. Plant. Biol..

[42] Seet B.T., Johnston J.B., Brunetti C.R., Barrett J.W., Everett H., Cameron C., Sypula J., Nazarian S.H., Lucas A., McFadden G. (2003). Poxviruses and immune evasion. Annu. Rev. Immunol..

[43] Yoo N.K., Pyo C.W., Kim Y., Ahn B.Y., Choi S.Y. (2008). Vaccinia virus-mediated cell cycle alteration involves inactivation of tumour suppressors associated with Brf1 and TBP. Cell Microbiol..

[44] Mo M., Fleming S.B., Mercer A.A. (2009). Cell cycle deregulation by a poxvirus partial mimic of anaphase-promoting complex subunit 11. Proc. Natl. Acad. Sci. USA.

[45] Postigo A., Martin M.C., Dodding M.P., Way M. (2009). Vaccinia-induced EGFR-MEK signalling and the anti-apoptotic protein F1L synergize to suppress cell death during infection. Cell Microbiol..

[46] McCart J.A., Ward J.M., Lee J., Hu Y., Alexander H.R., Libutti S.K., Moss B., Bartlett D.L. (2001). Systemic cancer therapy with a tumor-selective vaccinia virus mutant lacking thymidine kinase and vaccinia growth factor genes. Cancer Res..

[47] Knutson B.A., Oh J., Broyles S.S. (2009). Downregulation of vaccinia virus intermediate and late promoters by host transcription factor YY1. J. Gen. Virol..

[48] Rosales R., Harris N., Ahn B.Y., Moss B. (1994). Purification and identification of a vaccinia virus-encoded intermediate stage promoter-specific transcription factor that has homology to eukaryotic transcription factor SII (TFIIS) and an additional role as a viral RNA polymerase subunit. J. Biol. Chem..

[49] Wright C.F., Hubbs A.E., Gunasinghe S.K., Oswald B.W. (1998). A vaccinia virus late transcription factor copurifies with a factor that binds to a viral late promoter and is complemented by extracts from uninfected HeLa cells. J. Virol..

[50] Broyles S.S. (2003). Vaccinia virus transcription. J. Gen. Virol..

[51] Yoder J.D., Chen T.S., Gagnier C.R., Vemulapalli S., Maier C.S., Hruby D.E. (2006). Pox proteomics: Mass spectrometry analysis and identification of Vaccinia virion proteins. Virol. J..

[52] Chung C.S., Chen C.H., Ho M.Y., Huang C.Y., Liao C.L., Chang W. (2006). Vaccinia virus proteome: Identification of proteins in vaccinia virus intracellular mature virion particles. J. Virol..

[53] De Silva F.S., Moss B. (2008). Effects of vaccinia virus uracil DNA glycosylase catalytic site and deoxyuridinetriphosphatase deletion mutations individually and together on replication in active and quiescent cells and pathogenesis in mice. Virol. J..

[54] Leite F.G.G., Torres A.A., de Oliveira L.C., Soares-Martins J.A.S., Pereira A.C.T.C., Trindade G.S., Abrahão J.S., Kroon E.G., Ferreira P.C.P., Bonjardim C.A. (2017). c-Jun integrates signals from both MEK/ERK and MKK/JNK pathways upon vaccinia virus infection. Arch. Virol..

[55] Moss B. (2013). Poxvirus DNA replication. Cold Spring Harb. Perspect. Biol..

