# Supplementary material for: The Host Factor Early Growth Response Gene (EGR-1) Regulates Vaccinia virus Infectivity during Infection of Starved Mouse Cells
Source: Viruses. 2018 Mar 21;10(4):140. doi: 10.3390/v10040140 (PMC5923434; doi:10.3390/v10040140)
Supplement: Supplementary file 1 [file viruses-10-00140-s001.pdf]

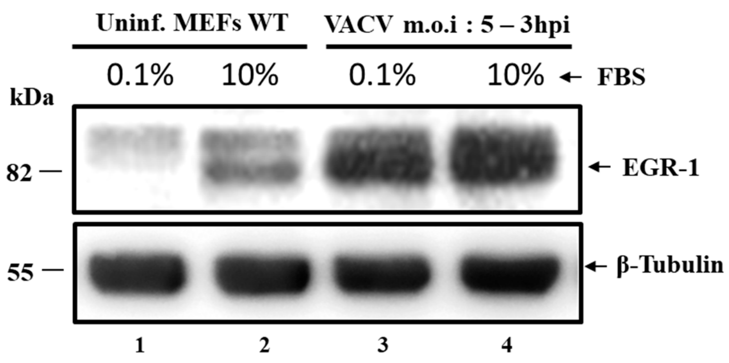

Supplementary Figure S1 – MEFs WT were grown in T-25 flasks and, where indicated, media was replaced with DMEM - FBS 0.1% for 12 hours to induce the starved state. Controls were kept under high FBS concentration. After 12 hours, cells were then infected or not with VACV-WR at an moi of 5. After 3 hours, infected and uninfected MEFs WT cells were collected and processed for Western blot analysis. Whole cell extracts were fractionated through 10% SDS-PAGE and transferred to PVDF membranes. Membranes were blotted with anti-EGR-1 antibody (1:1.500) or with anti-β-Tubulin, as loading control.

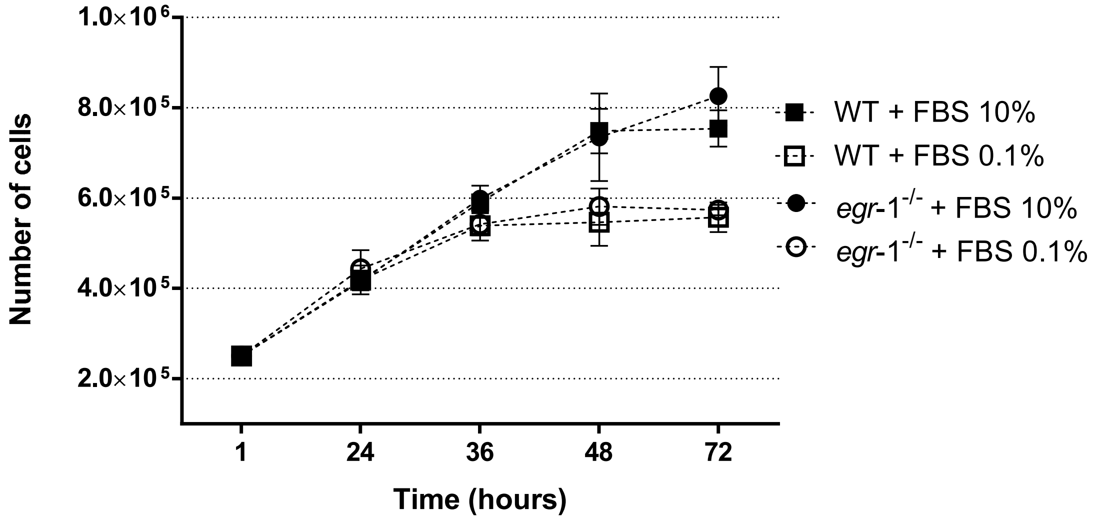

Supplementary Figure S2: 2.5x10^5 cells of both MEFs WT and *egr-1*$^{-/-}$ were seeded into each well of a 6-well plate using DMEM supplemented with 10% FBS. After 24 hours of incubation, cells were serum starved by replacing the media with DMEM containing 0.1% FBS, or were left growing under high FBS concentration (10%). After 12 hours, both serum starved and serum treated cells were individually counted using a hemocytometer. The cell population in each of the treatment groups was counted at 1, 24, 36, 48 and 72 hours after seeding. The graph plot represents the total number of cells in each group, at specific time

points. Population of serum-treated cells increased until the last time-point analyzed. The cell population numbers of serum-starved cells remained steady after 36 hours of seeding (12 hours of starvation), and even after 72 hours (48 hours of starvation) independent of the cell genotype. Experiment was performed in triplicate. The graph was plotted with Graphpad Prism 6.0.
